# Recombinant expression and biochemical characterization of a novel keratinase BsKER71 from feather degrading bacterium *Bacillus subtilis* S1-4

**DOI:** 10.1186/s13568-019-0939-6

**Published:** 2020-01-15

**Authors:** Bin Yong, Xueting Fei, Huanhuan Shao, Pan Xu, Youwen Hu, Weimin Ni, Qiuju Xiao, Xiang Tao, Xinyi He, Hong Feng

**Affiliations:** 1 College of Life Sciences, Sichuan Normal University, Chengdu, Sichuan 610101 China; 2 College of Life Sciences, Sichuan University, Chengdu, Sichuan 610064 China

**Keywords:** Feather degradation, Keratinase, BsKER71, *Bacillus subtilis* S1-4

## Abstract

*Bacillus subtilis* S1-4, isolated from chicken feather could efficiently degrade feathers by secreting several extracellular proteases. In order to get insight into the individual protease involved in keratin hydrolysis, a keratinase designed as BsKER71 was cloned and expressed in *Bacillus subtilis* WB600. In silico analysis revealed that BsKER71 protein contained a mature protein of 36.1 kDa. Further, purified BsKER71 could hydrolyze a variety of natural proteins, such as fibrous protein, collagen protein, casein, keratin and bovine serum albumin. In addition, this keratinase exhibited high enzyme activity in a wide range of pH and optimal pH of 10.0 and 9.0 in the hydrolysis of casein and keratin, respectively. Similarly, the optimal temperature was 55 °C and 50 °C for the hydrolysis of above two substrates, respectively. The hydrolytic activity was significantly inhibited by phenylmethanesulfonyl fluoride (PMSF), indicating the presence of serine residue in the active site. Moreover, ethylenediaminetetraacetic acid (EDTA) and phenanthroline moderately inhibited the hydrolytic activity. The catalytic activity was stimulated by Mg^2+^ and Ca^2+^, but greatly inhibited by Cu^2+^. Furthermore, several chemicals exhibited different effects on the hydrolysis of casein and keratin by BsKER71. These results provided a better understanding of BsKER71 from feather degrading bacterium *B. subtilis* S1-4.

## Introduction

Keratin is the key structural component of outer coat of feather, hair, nail, horn, hoof and skin of animals. It is extremely stable and resistant to degradation due to its specific molecular structure (Bradbury 1973). For instance, 7.3 cysteine residues are present per 100 residues in feather keratin (Sahni et al. 2015). The high proportion of cysteine is the result of high degrees of cross-linking for keratin protein. Therefore, keratin protein possesses resistance to digestion by proteolytic enzymes such as trypsin, pepsin, and papain (Kalaikumari et al. 2019). Keratin accounts for 90% of chicken feather in mass and 10% of total chicken weight (Brandelli 2008; Acda 2010; Tseng 2011), it was reported that about several million tons of chicken feathers are produced as waste by three major chicken producers (United States, China and Brazil) in the world at 2011 (Cedrola et al. 2012; Poopathi et al. 2014; Verma et al. 2016). The accumulation of huge amounts of feathers could result in serious environmental problems (Matikeviciene et al. 2009; Siano 2014), and transmission of various diseases, such as Marek’s disease, Creutzfeldt-Jacob, bird flu, and others (Sahni et al. 2015). Therefore, it is economical and environment-friendly to convert the feather into valuable protein sources. By using conventional processing, such as steam pressure, feathers can be converted into feather meal. However, conventional processing involved serial operations and several stages that promoted pollution and high energy consumption (Bouacem et al. 2016). The other disadvantage for conventional feather disposal methods is the loss of nutrients especially loss of essential amino acids such as lysine, methionine and tryptophan (Cai and Zheng 2009). Alternatively, microbial enzymes have been used for bio-processing of poultry waste, which provided an economic and environment-friendly strategy for the utilization of feather waste (Yusuf et al. 2015, 2016; Sanghvi et al. 2016). Enzyme proteases account for 40% of global market and industrial demand and are still in a growing trend. The current application of proteases generally requires consideration of appropriate specificity and stability of pH, temperature, surfactants, and organic solvents (Sanghvi et al. 2016). Therefore, the search for proteases with higher enzyme activity and milder conditions is urgently needed for industrial applications.

Keratinase is one of the proteolytic enzymes, which can hydrolyze insoluble feather keratins into free amino acids and polypeptides (Gupta et al. 2015). Currently, various keratinases have been purified or cloned from bacteria, yeasts, and fungi (Ramnani et al. 2005; More et al. 2013). However, keratinases from different sources are usually expressed and exhibited great diversity in biochemical and biophysical properties (Selvam and Vishnupriya 2012). At present, this enzyme has been widely used in cleaning sewage systems, food processing, textile and leather processing, medicine and cosmetics industries (Verma et al. 2017; Zhang et al. 2016). At present, a fairly large number of microbial sources with keratinolytic activity have been described from bacteria, yeasts, and fungi. Keratin-degrading nature has been reported for *Bacillus*, *Streptomyce*s, *Candida*, *Aspergillus* and so on (Vermelho et al. 2010). By fermentation, these microorganisms can hydrolyze feathers and produce different nutrients, especially various amino acids. Among these microorganisms, *Bacillus subtilis* strains are considered as effective keratin degraders that can secrete various enzymes, such as protease, amylase, and cellulose (Vlamakis et al. 2013). Therefore, it has been extensively studied to produce these enzymes at industrial scale (Buescher et al. 2012; Alponti et al. 2016). In addition, *B. subtilis* was recognized as a safe host bacterium to be used to produce industrial enzymes, and vaccine antigens and drugs (Zokaeifar et al. 2012; van Dijl and Hecker 2013). Previously, a new strain of *B. subtilis* S1-4 was isolated from chicken feather, which could secrete several extracellular proteases to hydrolyze various protein substrates, such as keratin, casein and gelatin, and efficiently degraded the chicken feathers (Yong et al. 2013a). In this study, a keratinase designed as BsKER71 was cloned and expressed in *Bacillus subtilis* WB600. Its potential for effective substrate degradation and enzymatic properties under different conditions was studied.

## Materials and methods

### Microbial strains

*Bacillus subtilis* S1-4, *B. subtilis* WB600 and *Escherichia coli* DH5α were used in this study. *B. subtilis* strain S1-4 was isolated from chicken feathers from a local poultry farm in China (Yong et al. 2013b). *B. subtilis* WB600 is an extracellular protease-deficient strain (*trpC2 nprA arp epr bpf mpr nprB*, CmR) and used as a host for recombinant expression of foreign gene. *E. coli* DH5α is used as a host for cloning.

### Sequence analyses of BsKER71

Sequence homologous searches were performed using BLASTP program (http://www.ncbi.nlm.nih.gov/BLAST) against the genome of *B. subtilis* S1-4 or GenBank (http://www.ncbi.nlm.nih.gov/). The isoelectric point (pI) and molecular weight prediction were determined by ProtParam (http://www.expasy.org/tools/protparam.html). Signal peptide was predicted at SignalP-4.1 Server (http://www.cbs.dtu.dk/services/SignalP/). PROSITE was used to analyze the active sites. 26 published amino acid sequences of keratinases from various species (*Bacillus licheniformis* MKU 3 (gi: 67866986), *Bacillus licheniformis* PWD-1 (gi: 998767), *Bacillus licheniformis* BBE11-1 (gi: 407280558), *Bacillus* sp. MKR1 (gi: 336462511), *Bacillus licheniformis* MKU 2 (gi: 67866984), *Bacillus licheniformis* S90 (gi: 358680691), *Bacillus licheniformis* RG1 (gi: 46277126), *Bacillus subtilis* BF20 (gi: 164654845), *Bacillus mojavensis* (gi: 50363121), *Bacillus licheniformis* ER-15 (gi: 351000221), *Bacillus licheniformis* RPk (gi: 169883790), *Bacillus licheniformis* N5 (gi: 656355213), *Bacillus licheniformis* UTM107 (gi: 823327614), *Bacillus licheniformis* MZK05 (gi: 668730483), *Bacillus licheniformis* DS23 (gi: 300390464), *Bacillus pumilus* A1 (gi: 222353760), *Bacillus pumilus* KS12 (gi: 300429856), *Bacillus tequilensis* Q7 (gi: 846451730), *Bacillus circulans* DZ100 (gi: 511676716), *Bacillus amyloliquefaciens* K11 (gi: 893694191), *Bacillus velezensis* KJN-2 (gi: 443298521), *Bacillus subtilis* Egy-Ker (gi: 973694715), *Bacillus subtilis* Egy-KerM (gi: 973694712), *Bacillus subtilis* RSE163 (gi: 727929352), *Bacillus subtilis* B-3 (gi: 336109555), *Bacillus subtilis* YYW-1 (gi: 164664938)) were retrieved from GenBank database. Multiple sequence alignment was constructed using ClustalW program (Chenna et al. 2013), and then neighbor-joining tree was constructed using MEGA6.0 program (Tamura et al. 2013).

### Cloning, expression and purification

Based on the genome of *B. subtilis* S1-4, a specific pair of primers was designed, 3371-F1 (5′-TGGACTGCAGTTCATCTCATTTCTTCCTCCC-3′) and 3371-R1 (5′-ATTCGGTACCGGAACATCAGGATGCTGAC-3′). The PCR reaction mixture (50 μL) contained 400 nmol/L of each primer, 100 μmol/L dNTP, 50 ng gDNA of S1-4, and Pfu DNA polymerase. The amplification conditions were as follows: initial denaturation at 95 °C for 2 min, 30 cycles at 94 °C for 30 s, 52 °C for 30 s, 68 °C for 2 min, and a final extension at 72 °C for 10 min. The PCR products were then purified using SanPrep DNA gel extraction kit (Sangon, Shanghai, China) following the manufacturer’s instructions, and digested by PstI and KpnI restriction enzymes (TaKaRa, Dalian, China). The resultant PCR products were cloned into *E. coli*–*B. subtilis* shuttling vector pSUGV4 digested with same restriction enzymes by DNA ligation and transformation. The resulting recombinant plasmid pSUGV4-KER71 was confirmed by PCR amplification and DNA sequencing.

The recombinant plasmid pSUGV4-KER71 was then transformed into *B. subtilis* WB600 as previously described (Shao et al. 2015). Several colonies were selected and inoculated onto Luria–Bertani (LB) agarose plates with skim milk (10 g/L), and incubated at 37 °C. The expression of recombinant BsKER71 was initially confirmed by the formation of hydrolytic circle. Then, a single colony was inoculated into 2 mL of LB broth, and incubated overnight at 37 °C. The overnight culture was inoculated in 2 L of LB broth containing 20 g skim milk, and kept in shaking condition at 220 rpm for 48 h to express the recombinant proteins. Cell-free supernatant was collected by centrifugation at 4 °C, to which ammonium sulfate was added at 60% saturation to precipitate the proteins. The precipitated protein was dissolved in 20 mmol/L of phosphate buffer (pH 8.0). Finally, the protein sample was loaded onto gel filtration column (Hiprep 16/60 Sephacryl S-200 High Resolution, GE Health Co., USA) and eluted at a rate of 2 mL/min with 20 mmol/L phosphate buffer (pH 8.0) on AKAT Primer System (GE Health Co., USA). The protease activity was determined by loading 10 μL from the fractions (2 mL) onto the skim milk-containing agarose plates. Then, the fractions that showed protease activity were pooled and dialyzed against 20 mmol/L phosphate buffer (pH 8.0). Subsequently, the concentration and purity of purified proteins were evaluated by sodium dodecyl sulfate–polyacrylamide gel electrophoresis (SDS-PAGE) using bovine serum albumin (BSA) as standard.

### Activity assay

To determine the hydrolytic reaction of selected natural proteins, 5 μL of gelatin (Solarbio, Beijing, China), fibrin (Solarbio, Beijing, China), collagen (Sigma Chemical Co., St. Louis, USA), BSA (Solarbio, Beijing, China), or casein (10 mg/mL) (AOBOX, Beijing, China) was mixed with 5 μL (4.8 μg) of purified BsKER71 in 20 μL boric acid–NaOH buffer (pH 9.6). After incubation for 20 min at 50 °C, proteolytic reaction was terminated by heating at 95 °C for 10 min. Then, samples were directly loaded onto 12% SDS-PAGE.

The keratinase activity was determined as previously described using keratin powder (XABC biotech Co., Xian, China) as substrate (Wawrzkiewicz et al. 1987). One unit of keratinolytic activity was defined as the amount of enzyme required to generate an increase of 0.01 OD value at 280 nm.

Similarly, caseinolytic activity was determined using previously described method (Wan et al. 2009). Folin–Ciocalteau reagent was purchased from Sangon Co. (Shanghai, China). One unit of caseinolytic activity was defined as the amount of enzyme required to produce 1 μg of tyrosine per min.

### Effect of pH and temperature on hydrolytic activity

The effect of pH on purified S-3371 activity was examined in the pH ranging from 5.0 to 14.0 using various buffer solutions: citric acid and sodium citrate buffer for 5.0, dibasic sodium phosphate buffer for 6.0–8.0, sodium hydroxide and glycine-sodium hydroxide buffer for 9.0–10.0, dibasic sodium phosphate and sodium hydroxide buffer for 11.0, potassium chloride and sodium hydroxide for 12.0–14.0. Hydrolytic activity of purified BsKER71 was performed at various pH described as above using casein and keratin as substrate.

Under optimal pH, effect of different temperatures (30–70 °C) on the catalytic activity was examined as described above using casein and keratin as substrate. To determine thermal stability, enzyme sample was diluted in boric acid–NaOH buffer (pH 9.6), and incubated at selected temperatures for 20 min. Each enzyme sample was immediately transferred on ice. Residual activity was determined as described above using casein and keratin as substrate.

### Effect of protease inhibitors, metal ions and chemical reagents

To determine the effect of protease inhibitors (EDTA, PMSF, Phenanthroline), metal ions (Zn^2+^, Co^2+^, Mg^2+^, Ca^2+^, Mn^2+^, Ni^2+^, Cu^2+^), and chemical reagents (SDS, DTT, Triton-100, Na_2_SO_3_, DMSO) on the hydrolytic activity of BsKER71, each compound at the indicated concentration was added to assaying mixture. The hydrolytic reaction was performed following the standard procedure as described above. All assays were performed in triplicates.

## Results

### Sequence analysis of keratinase-encoding gene (*Bsker71*)

**Fig. 1:**
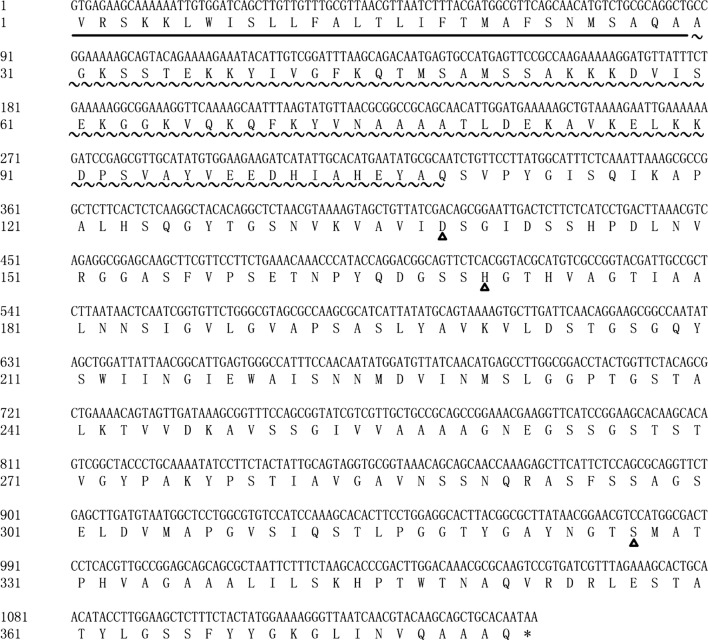
The nucleotide and protein sequence of BsKER71. The underline indicates signal peptide, wave line mean peptidase inhibitor I9 domain, and triangles mean active sites

In this study, the published keratinase sequences (gi: 998767 from *B. licheniformis* and gi: 336109555 from *B. subtilis*) were aligned with the genomic data of the *B. subtilis* S1-4 by local BLASTP. A homologous sequence was identified in the genome of S1-4. The entire coding sequence (designed as *Bsker71*) was 1146 bp in length (accession number: MN256128), encoding a deduced protein of 381 amino acids with a molecular weight of 39.45 kDa and theoretical isoelectric point of 9.04 (Fig. 1). Hydrophobicity analysis showed that BsKER71 protein might be a hydrophilic protein for its Grand average of hydropathicity (GRAVY) of 0.054 by ProtParam. Further, three active sites were found in the amino acid sequence, Asp138, His170, and Ser327, suggesting that this enzyme might belong to serine protease. Signal peptide analysis demonstrated that BsKER71 protein contained a predicted signal peptide sequence (1–29 aa), followed by a prepeptide (30–108 aa). Therefore, this sequence was predicted to encode a secretory keratinase.

**Fig. 2:**
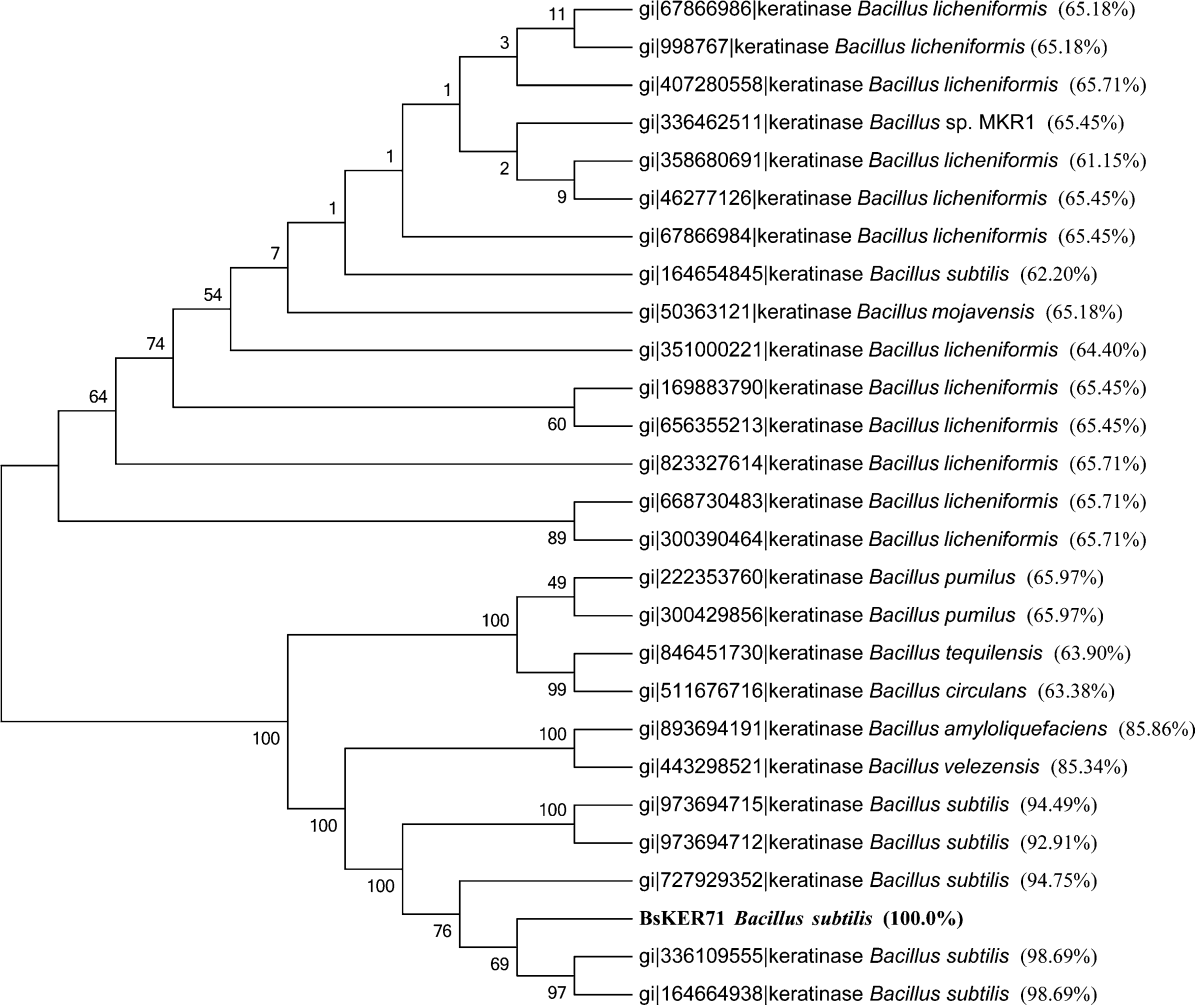
Clustering analyses of BsKER71 protein with published keratinases

The results of multiple sequence alignment and neighbor-Joining tree showed that BsKER71 was grouped most closely to KerC of *B. subtilis* B-3 (gi: 336109555) and *B. subtilis* YYW-1 (gi: 164664938), but appeared to be distinct from keratinase (e.g. gi: 998767) of *B. licheniformis* PWD-1 (Fig. 2). These results indicated that *BsKER71* might encode keratinase.

### Cloning and expression of BsKER71 protein

Based on the nucleotide sequence of *Bsker71*, entire coding sequence and 5′-upstream was cloned into *E.coli*–*B. subtilis* shuttling vector pSUGV4 that was constructed based on pUB110 and used to express exogenous protein (Liu et al. 2001), resulting in a recombinant plasmid pSUGV4-KER71. After confirmation by DNA sequencing, the recombinant plasmid was transformed into *B. subtilis* WB600, in which the expression of *Bsker71* gene was determined by its native promoter. Initially, the expression of keratinase was determined on skim milk-containing plate. Afterwards, positive WB600 recombinants were inoculated into fermentation medium to produce keratinase.

Recombinant keratinase was purified from the supernatant through ammonium sulfate precipitation and gel filtration chromatography column. A typical elution profile was obtained that showed a peak (peak 2) (Additional file 1: Fig. S1). The keratinase activity of this peak was confirmed by loading the eluted samples onto skim milk-containing agarose plates (Additional file 2: Fig. S2). Finally, the recombinant enzyme sample was concentrated and its concentration and purity were evaluated by SDS-PAGE using bovine serum albumin (BSA) as standard (Additional file 3: Fig. S3). The concentration of purified protein was estimated to be 0.96 μg/μL using Quantity One version 4.6.2.

### Hydrolysis of natural protein substrates

**Fig. 3:**
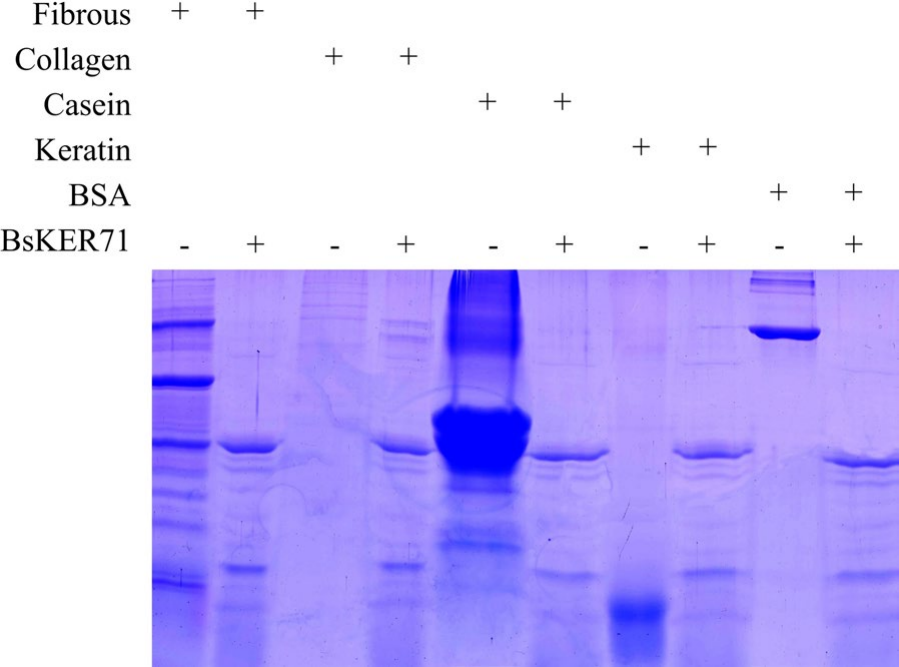
Hydrolysis of natural protein substrates by the purified BsKER71. From left to right represented the following lane: fibrous, fibrous treated by BsKER71, collagen, collagen treated by BsKER71, casein, casein treated by BsKER71, keratin, keratin treated by BsKER71, BSA, BSA treated by BsKER71

To investigate various substrates, five natural proteins, such as fibrous protein, collagen protein, casein, keratin and bovine serum albumin were used as substrates. The molecular weight of the natural protein is significantly reduced, especially for casein (Fig. 3). In addition, BsKER71 also exhibited pretty good degradation of keratin. The results indicated that BsKER71 could perform hydrolysis to several natural protein substrates and hydrolyzed them into small molecular substances.

### Effect of pH and temperature on the hydrolytic activity

**Fig. 4:**
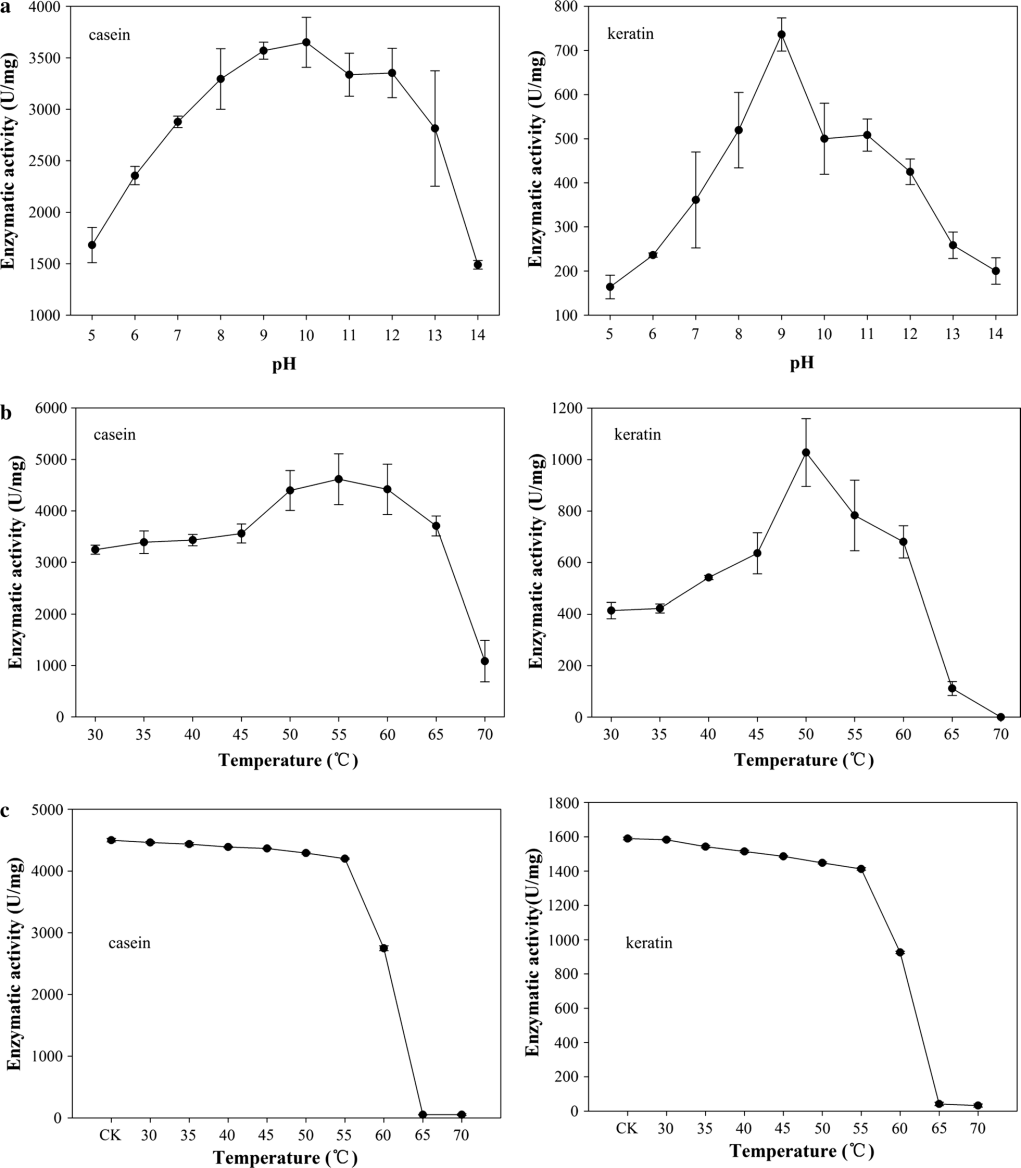
Effects of pH, temperature and thermal stability on hydrolysis of casein and keratin by BsKER71. **a** Effects of pH (4.0–14.0) on the enzymatic activity for BsKER71 with two kinds of substrate (left: casein; right: keratin); **b** effect of temperature on the enzymatic activity for BsKER71 with two kinds of substrate (left: casein; right: keratin); **c** the thermal stability of BsKER71 at different temperature with two kinds of substrate (left: casein; right: keratin)

Figure 4a shows that BsKER71 could hydrolyze different protein substrates in a wide range of pH (4.0–14.0). However, the effect of pH on the hydrolytic activity was slightly different with respect to various substrates. The optimal pH for casein and keratin were 10.0 (p < 0.01) and 9.0 (p < 0.01), respectively. BsKER71 exhibited high enzyme activity in a wide range of pH and higher enzymatic activity for casein compared to keratin.

The effect of temperature on the hydrolytic activity of BsKER71 was shown in Fig. 4b. For casein as substrate, BsKER71 exhibited higher hydrolytic activity from 50 to 60 °C with maximum activity at 55 °C (4616.67 ± 387.87 U/mg, p < 0.01). When keratin was used as substrate, BsKER71 exhibited maximum activity at 50 °C (p < 0.01), which dropped over 60 °C. These results showed that BsKER71 has a narrow temperature range for hydrolytic activity when keratin was used as substrate than casein.

In addition, the thermal stability of BsKER71 was investigated. Results indicated that the enzyme was more stable below 55 °C without great loss of the residue activity irrespective of the substrate used (casein or keratin). When the temperature was above 55 °C, hydrolytic activity was rapidly decreased (Fig. 4c).

### Effect of keratinase inhibitor, metal ions and chemical reagents on the hydrolytic activity

**Fig. 5:**
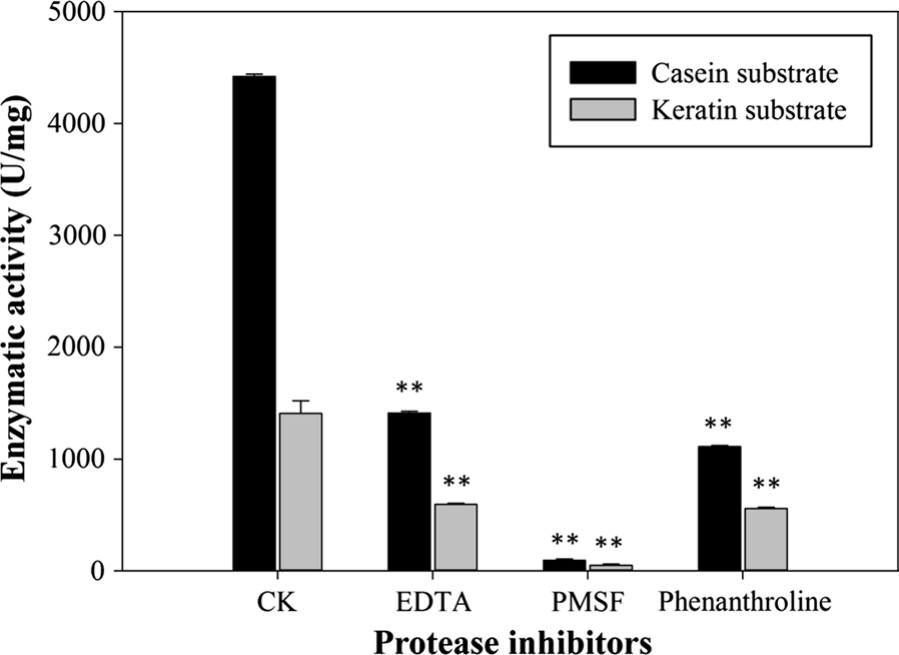
Effects of three protease inhibitors on protease activity of BsKER71 with two kinds of substrate (casein and keratin). From left to right represented the following treatment for BsKER71: CK, EDTA, PMSF, phenanthroline

It was observed that PMSF almost completely inhibited the hydrolytic activity, indicating that BsKER71 is a serine protease (Fig. 5). However, EDTA and phenanthroline exhibited moderate inhibition (about 60%) on the hydrolytic activity, suggesting that the catalytic reaction of BsKER71 might require transition metal ion.

The effect of metal ions on the catalysis of BsKER71 was shown in Table 1. It was found that Mg^2+^ (2 mmol/L) and Ca^2+^ (2 mmol/L) promoted the hydrolytic activity of BsKER71 to some extent when casein and keratin were used as substrates. In contrast, Cu^2+^ (2 mmol/L) almost completely inhibited the enzymatic activity. The other metal ions exhibited only slight inhibition, maintaining > 90% activity. However, no obvious difference was observed for both substrates.

**Table 1:** Effect of various metal ions on hydrolytic activity of BsKER71

Metal ions	Concentration (mmol/L)	Hydrolytic activity of different substrate	
		Casein (%)	Keratin (%)
CK	0	100.00	100.00
Zn^2+^	2	97.00	95.48
Co^2+^	2	93.73	92.70
Mg^2+^	2	114.73	112.13
Ca^2+^	2	116.64	115.81
Mn^2+^	2	94.71	89.45
Ni^2+^	2	95.36	94.21
Cu^2+^	2	2.78	1.14

Subsequently, several surfactants were selected to investigate their effect on enzymatic activity. For casein as substrate, the hydrolytic activity of BsKER71 was slightly enhanced by 1 mmol/L of DMSO, DTT, Triton-100 and SDS, but slightly inhibited by 1 mmol/L Na_2_SO_3_. With the increased concentration of the surfactants to 5 mmol/L, slight inhibition was observed, especially 5 mmol/L of DMSO exhibited significant inhibition (Table 2). For keratin as substrate, the hydrolytic activity of BsKER71 was slightly inhibited by 1 mmol/L of Triton-100 and SDS, and 5 mmol/L of DTT. When 5 mmol/L of SDS, Na_2_SO_3_ and DMSO were used, no keratin-hydrolytic activity was observed. Overall, these reagents showed different effects on the hydrolytic activity of BsKER71 to various substrates at different concentrations.

**Table 2:** Effect of chemical reagents on hydrolytic activity of BsKER71

Surfactant	Casein as substrate		Keratin as substrate	
	1 mmol/L	5 m mol/L	1 mmol/L	5 mmol/L
CK	100.00	100.00	100.00	100.00
SDS	103.74	97.12	86.28	0.00
DTT	103.74	96.92	100.87	91.55
Triton-100	107.72	98.37	80.07	103.66
Na_2_SO_3_	93.63	98.99	101.38	0.00
DMSO	107.53	16.17	103.70	0.00

## Discussion

Enzymatic degradation of feathers provides an attractive method for degradation of keratin to value-added products like amino acids, nitrogen fertilizers or feed supplements which can be used as feed supplement or organic fertilizer (Bhari et al. 2018). Keratinases can effectively degrade keratin so that they replace the conventional pollution-creating physicochemical methods, bestow shrink-resistance and improve handling properties (Lv et al. 2010). A new *B. subtilis* strain S1-4 was isolated from chicken feathers, which could secrete several keratinases to efficiently degrade the feathers. In order to get insights into extracellular proteases, especially those involved in feather degradation, a protease encoding gene *Bsker71* was cloned, expressed and biochemically characterized from *B. subtilis* S1-4. Using multiple sequence alignment of amino acid sequences with other keratinases and substrate specificity of hydrolysis, this protein was confirmed as keratinase. Bioinformatics analysis indicated that BsKER71 might be an extracellular enzyme with a deduced signal peptide and propeptide, which was confirmed in heterologous expression in *B. subtilis* WB600. With respect to molecular weight, BsKER71 was similar to that from *Bacillus subtilis* (39.5 kDa) (Hou et al. 2012), *Bacillus amyloliquefaciens* (39.14 kDa) (Yang et al. 2015) and *Bacillus circulans* (39.45 kDa) (Benkiar et al. 2013). However, it was different from those from various fungi, such as *Scopulariopsis brevicaulis* (39 kDa) (Anbu et al. 2005), *Trichophyton mentagrophytes* (38 kDa) (Muhsin and Aubaid 2001), and *Aspergillus parasiticus* (36 kDa) (Anitha and Palanivelu 2013).

The purified BsKER71 keratinase exhibited wide substrate specificity, and could degrade various natural proteins, such as fibrin, collagen, casein, keratin and BSA. Therefore, it was suggested that BsKER71 has certain potential in environment-friendly dehairing process, and bioconversion of keratinous wastes (Tork et al. 2013). Besides, purified BsKER71 showed a wide adaptation in pH and temperature, with the optimal pH 9.0–10.0 and temperature 50–55 °C for casein and keratin, respectively. This was consistent with other keratinases from various microbes (Brandelli et al. 2010; Kuo et al. 2012; Anitha and Palanivelu 2013). It was noteworthy that the optimal pH 9.0 for keratin hydrolysis of BsKER71 was higher than the pH 8.0 to achieve efficient degradation of feather by *B. subtilis* S1-4.

The keratinase BsKER71 reported in this study was strongly inhibited by PMSF, a well-known serine protease inhibitor, indicating that BsKER71 might be a serine protease. In fact, a serine residue was predicted to be involved in the active site. Similar results were observed from other reported keratinases of *Doratomyces microspores* (Gradišar et al. 2000), *Chryseobacterium* sp. (Brandelli 2005), and *A. parasiticus* (Anitha and Palanivelu 2013). Further, EDTA and phenanthroline also inhibited the catalytic activity of BsKER71, suggesting that it might require transition metal for hydrolysis. These results were in agreement with previous studies, which showed that serine proteases could be affected by metalloprotease inhibitors (Ramnani and Gupta 2004).

Various studies have reported that the keratinase activity can be stimulated by divalent metal ions, such as Mg^2+^ and Ca^2+^, and might play an important role in maintaining the conformation of the enzyme complex as salts or ion bridges (Riffel et al. 2003). The increase of keratinase activity in the presence of Ca^2+^ is a typical characteristic of serine proteases. This phenomenon was also observed with BsKER71 in this study, which further confirmed that BsKER71 could be a serine protease. However, Mn^2+^ slightly inhibited the hydrolytic activity of BsKER71, which is in contrast to other reported keratinases from *B. megaterium*, *A. parasiticus*, *Bacillus* sp. L4 and *Bacillus subtilis* K-5 (Zhang et al. 2009; Rajesh et al. 2010; Anitha and Palanivelu 2013; Singh et al. 2014). Furthermore, Zn^2+^, Co^2+^, Cu^2+^, and Ni^+^ inhibited the enzymatic activity of BsKER71 at different extent, especially Cu^2+^. Similar observation was reported in other fungal and bacterial keratinases, such as purified keratinases from *Trichoderma atroviridae* and *Streptomyces* sp. (Tatineni et al. 2008; Cao et al. 2008).

It has been reported that the addition of DTT resulted in increased keratinase activity through the breakage of disulfide bonds in tightly packed keratins (Riffel et al. 2003; Gradisar et al. 2005). It was found that 5 mmol/L of DTT significantly increased the keratinase activities of *P. marquandii* and *D. microspores*. In contrast, in this study, the enzymatic activity of BsKER71 was slightly inhibited (5 mmol/L) or increased (1 mmol/L) by DTT. In addition, it was observed that DTT might have almost no influence on the enzyme activity of BsKER71. This was similar to that of keratinase KERAB from *Streptomyces* sp. strain AB1 (Jaouadi et al. 2010). With respect to SDS, higher concentrations could inhibit the keratinase activity of BsKER71, which was consistent with that from *P. marquandii* and *D. microspores* (Gradisar et al. 2005). According to another study, keratinases were inhibited, stimulated or stable in the presence of DTT or SDS, indicating that different non-ionic detergents and solvents showed distinctive effects to different keratinase Gupta and Ramnani (2006).

In conclusion, a novel keratinase BsKER71 from *B. subtilis* S1-4 was cloned, expressed and characterized in this study. The purified enzyme could hydrolyze several natural proteins and exhibited temperature adaptability and higher enzyme activity at alkaline pH. Results indicated that BsKER71 had a certain potential in safe, economical and environment-friendly utilization of feather wastes.

## Supplementary information

**Additional file 1: Figure S1.** A typical elution profile of the recombinant keratinase by gel filtration chromatography column. Peak 1: unknown protein; Peak 2: BsKER71 protein; Red peak: ammonium sulfate.

**Additional file 2: Figure S2.** The protease activity test of eluting samples from peak 2 on the milk containing agarose plate.

**Additional file 3: Figure S3.** Quantitative determination of BsKER71 protein on SDS-PAGE. Lane 1, 2, 3, 4, 5 correspond to 0.5, 1.0, 2.0, 3.0, and 4.0 μg BSA, respectively; lane 6 and 7 were loaded with 2.5 μL and 5.0 μL purified BsKER71 protein.

## Data Availability

The *Bsker71* gene sequence of *Bacillus subtilis* S1-4 that was used in this research have been submitted to NCBI Genbank database with the accession number MN256128.

## References

[CR1] Acda MN (2010). Waste chicken feather as reinforcement in cement-bonded composites. Philipp J Sci.

[CR2] Alponti JS, Maldonado RF, Ward RJ (2016). Thermostabilization of *Bacillus subtilis* GH11 xylanase by surface charge engineering. Int J Biol Macromol.

[CR3] Anbu P, Gopinath SCB, Hilda A, Annadurai G (2005). Purification of keratinase from poultry farm isolate *Scopulariopsis brevicaulis* and statistical optimization of enzyme activity. Enzyme Microb Tech.

[CR4] Anitha TS, Palanivelu P (2013). Purification and characterization of an extracellular keratinolytic protease from a new isolate of *Aspergillus parasiticus*. Protein Expr Purif.

[CR5] Benkiar A, Nadia ZJ, Badis A, Rebzani F, Soraya BT, Rekik H, Naili B, Ferradji FZ, Bejar S, Jaouadi B (2013). Biochemical and molecular characterization of a thermo-and detergent-stable alkaline serine keratinolytic protease from *Bacillus circulans* strain DZ100 for detergent formulations and feather-biodegradation process. Int Biodeter Biodegrad.

[CR6] Bhari R, Kaur M, Singh RS, Pandey A, Larroche C (2018). Bioconversion of chicken feathers by *Bacillus aerius* NSMk2: a potential approach in poultry waste management. Bioresour Technol Rep.

[CR7] Bin Y, Yang BQ, Feng H (2013). Efficient degradation of raw chicken feather into soluble peptides and free amino acids by a newly isolated *Bacillus subtilis* S1-4. Res J Biotechnol.

[CR8] Bouacem K, Bouanane-Darenfed A, Jaouadi NZ, Joseph M, Hacene H, Ollivier B, Fardeau ML, Bejar S, Jaouadi B (2016). Novel serine keratinase from *Caldicoprobacter algeriensis* exhibiting outstanding hide dehairing abilities. Int J Biol Macromol.

[CR9] Bradbury J.H. (1973). The Structure and Chemistry of Keratin Fibers. Advances in Protein Chemistry.

[CR10] Brandelli A (2005). Hydrolysis of native proteins by a keratinolytic protease of *Chryseobacterium* sp. Ann Microbiol.

[CR11] Brandelli A (2008). Bacterial keratinases: useful enzymes for bioprocessing agroindustrial wastes and beyond. Food Bioprocess Tech.

[CR12] Brandelli A, Daroit DJ, Riffel A (2010). A Biochemical features of microbial keratinases and their production and applications. Appl Microbiol Biot.

[CR13] Buescher JM, Liebermeister W, Jules M, Uhr M, Muntel J, Botella E, Hessling B, Kleijn RJ, Le Chat L, Lecointe F (2012). Global network reorganization during dynamic adaptations of *Bacillus subtilis* metabolism. Science.

[CR14] Cai C, Zheng X (2009). Medium optimization for keratinase production in hair substrate by a new *Bacillus subtilis* KD-N2 using response surface methodology. J Ind Microbiol Biotechnol.

[CR15] Cao L, Tan H, Liu Y, Xue X, Zhou S (2008). Characterization of a new keratinolytic *Trichoderma atroviride* strain F6 that completely degrades native chicken feather. Lett Appl Microbiol.

[CR16] Cedrola SML, de Melo ACN, Mazotto AM, Lins U, Zingali RB, Rosado AS, Peixoto RS, Vermelho AB (2012). Keratinases and sulfide from *Bacillus subtilis* SLC to recycle feather waste. World J Microbiol Biotechnol.

[CR17] Chenna R, Sugawara H, Koike T, Lopez R, Gibson TJ, Higgins DG, Thompson JD (2013). Multiple sequence alignment with the clustal series of programs. Nucleic Acids Res.

[CR18] Gradisar H, Friedrich J, Krizaj I, Jerala R (2005). Similarities and specificities of fungal keratinolytic proteases:comparison of keratinases of *Paecilomyces marquandii* and *Doratomyces microsporus* to some known proteases. Appl Environ Microbiol.

[CR19] Gradišar H, Kern S, Friedrich J (2000). Keratinase of *Doratomyces microsporus*. Appl Microbiol Biotechnol.

[CR20] Gupta R, Ramnani P (2006). Microbial keratinases and their prospective applications:an overview. Appl Microbiol Biotechnol.

[CR21] Gupta S, Nigam A, Singh R (2015). Purification and characterization of a *Bacillus subtilis* keratinase and its prospective application in feed industry. Acta Biol Szeged.

[CR22] Hou SQ, Wang LH, Lai X, Chen H, Wu Q, Han X (2012). Isolation, identification of B-3 *Bacillus subtilis* and cloning, expression of kerC. China Env Sci.

[CR23] Jaouadi B, Abdelmalek B, Fodil D, Ferradji FZ, Rekik H, Zaraî N, Bejar S (2010). Purification and characterization of a thermostable keratinolytic serine alkaline proteinase from *Streptomyces* sp. strain AB1 with high stability in organic solvents. Bioresour Technol.

[CR24] Kalaikumari SS, Vennila T, Monika V, Chandraraj K, Gunasekaran P, Rajendhran J (2019). Bioutilization of poultry feather for keratinase production and its application in leather industry. J Cleaner Prod.

[CR25] Kuo JM, Yang JI, Chen WM, Pan MH, Tsai ML, Lai YJ, Hwang A, Pan BS, Lin CY (2012). Purification and characterization of a thermostable keratinase from *Meiothermus* sp. I40. Int Biodeter Biodegrad.

[CR26] Leffv LX, Sim MH, Li YD, Min J, Feng WH, Guan WJ, Li YQ (2010). Production, characterization and application of a keratinase from *Chryseobacterium* sp. nov.. Proc Biochem.

[CR27] Liu CJ, Qing H, Zhao L, Zhang YZ (2001). Construction of a *Escherichia coli*–*Bacillus subtilis* shuttle vector pSUGV4. J Sichuan Univ.

[CR28] Matikeviciene V, Masiliuniene D, Grigiskis S (2009) Degradation of keratin containing wastes by bacteria with keratinolytic activity, Environment Technology. In: Resources proceedings of the 7th international scientific and practical conference, Rezekne, 2009

[CR29] More SS, Lakshmi Sridhar D, Prakash SN, Vishwakarma J, Umashankar S (2013). Purification and properties of a novel fungal alkaline keratinase from *Cunninghamella echinulata*. Turk J Biochem.

[CR30] Muhsin TM, Aubaid AH (2001). Partial purification and some biochemical charactcristics of exocellular keratinase from *Trichophyton mentagrophytes* var. erinacei. Mycopathologia.

[CR31] Poopathi S, Thirugnanasambantham K, Mani C, Lakshmi PV, Ragul K (2014). Purification and characterization of keratinase from feather degrading bacterium useful for mosquito control—a new report. Trop Biomed.

[CR32] Rajesh T, Deepa D, Divya D, Mahesh M, Somshekar S (2010). Isolation, purification, characterization and applications of serine protease from *Bacillus megaterium*. J Appl Nat Sci.

[CR33] Ramnani P, Gupta R (2004). Optimization of medium composition for keratinase production on feather by *Bacillus licheniformis* RG1 using statistical methods involving response surface methodology. Biotechnol Appl Biochem.

[CR34] Ramnani P, Singh R, Gupta R (2005). Keratinolytic potential of *Bacillus licheniformis* RG1: structural and biochemical mechanism of feather degradation. Can J Microbiol.

[CR35] Riffel A, Lucas F, Heeb P, Brandelli A (2003). Characterization of a new keratinolytic bacterium that completely degrades native feather keratin. Arch Microbiol.

[CR36] Sahni N, Sahota PP, Phutela UG (2015). Bacterial keratinases and their prospective applications:a review. Int J Curr Microbiol Appl Sci.

[CR37] Sanghvi G, Patel H, Vaishnav D, Oza T, Dave G, Kunjadia P, Sheth N (2016). A novel alkaline keratinase from *Bacillus subtilis* DP1 with potential utility in cosmetic formulation. Int J Biol Macromol.

[CR38] Selvam K, Vishnupriya B (2012). Biochemical and molecular characterization of microbial keratinase and its remarkable applications. Turk J Biochem.

[CR39] Shao H, Cao Q, Zhao H, Tan X, Feng H (2015). Construction of novel shuttle expression vectors for gene expression in *Bacillus subtilis* and *Bacillus pumilus*. J Med Vet Mycol.

[CR40] Siano N (2014) Reprogramming of energy metabolism by oncogenic Marek’s disease virus (MDV) in chicken embryo fibroblasts (CEFs). Dissertation, University of Delaware

[CR41] Singh S, Gupta P, Sharma V, Koul S, Kour K, Bajaj BK (2014). Multifarious potential applications of keratinase of *Bacillus subtilis* K-5. Biocatal Biotransfor.

[CR42] Tamura K, Stecher G, Peterson D, Filipski A, Kumar S (2013). MEGA6: molecular evolutionary genetics analysis version 6.0. Mol Biol Evol.

[CR43] Tatineni R, Doddapaneni KK, Potumarthi RC, Vellanki RN, Kandathil MT, Kolli N, Mangamoori LN (2008). Purification and characterization of an alkaline keratinase from *Streptomyces* sp. Bioresour Technol.

[CR44] Tork SE, Shahein YE, El-Hakim AE, Abdel-Aty AM, Aly MM (2013). Production and characterization of thermostable metallo-keratinase from newly isolated *Bacillus subtilis* NRC 3. Int J Biol Macromol.

[CR45] Tseng FCJ (2011) Biofibre production from chicken feather. Dissertation, University of Waikato

[CR46] van Dijl J, Hecker M (2013). *Bacillus subtilis*: from soil bacterium to super-secreting cell factory. Microbiol Cell Fact.

[CR47] Verma A, Singh H, Anwar S, Chattopadhyay A, Tiwari KK, Kaur S, Dhilon GS (2016). Microbial keratinases: industrial enzymes with waste management potential. Crit Rev Biotechnol.

[CR48] Verma A, Singh H, Anwar S, Chattopadhyay A, Tiwari KK, Kaur S, Dhilon GS (2017). Microbial keratinases: industrial enzymes with waste management potential. Crit Rev Biotechnol.

[CR49] Vermelho AB, Mazotto AM, de Melo ACN, Vieira FHC, Duarte TR, Macrae A, Nishikawa MM, da Silva Bon EP (2010). Identification of a *Candida parapsilosis* strain producing extracellular serine peptidase with keratinolytic activity. Mycopathologia.

[CR50] Vlamakis H, Chai Y, Beauregard P, Losick R, Kolter R (2013). Sticking together: building a biofilm the *Bacillus subtilis* way. Nat Rev Microbiol.

[CR51] Wan MY, Wang HY, Zhang YZ, Feng H (2009). Substrate specificity and thermostability of the dehairing alkaline protease from *Bacillus pumilus*. Appl Biochem Biotech.

[CR52] Wawrzkiewicz K, Łobarzewski J, Wolski T (1987). Intracellular keratinase of *Trichophyton gallinae*. J Med Vet Mycol.

[CR53] Yang L, Wang H, Lv Y, Bai Y, Luo H, Shi P, Huang H, Yao B (2015). Construction of a rapid feather-degrading bacterium by overexpression of a highly efficient alkaline keratinase in its parent strain *Bacillus amyloliquefaciens* K11. J Agr Food Chem.

[CR60] Yong B, Yang BQ, Feng H (2013). Eicient degradation of raw chicken feather into soluble peptides and free amino acids by a newly isolated *Bacillus subtilis* S1–4. Res J Biotechnol.

[CR54] Yong B, Yang BQ, Zhao CW, Feng H (2013). Draft genome sequence of *Bacillus subtilis* strain S1-4, which degrades feathers efficiently. Genome Announc.

[CR55] Yusuf I, Shukor MY, Syed MA, Yee PL, Shamaan NA, Ahmad SA (2015). Investigation of keratinase activity and feather degradation ability of immobilised *Bacillus* sp. Khayat in the presence of heavy metals in a semi continuous fermentation. J Chem Pharm Sci.

[CR56] Yusuf I, Ahmad SA, Phang LY, Syed MA, Shamaan NA, Khalil KA, Dahalan FA, Shukor MY (2016). Keratinase production and biodegradation of polluted secondary chicken feather wastes by a newly isolated multi heavy metal tolerant bacterium-*Alcaligenes* sp. AQ05-001. J Environ Manag.

[CR57] Zhang Q, Sun D, Yang W, Liu Q, Bai F (2009). Enzymatic characteristics of keratinase from *Bacillus* sp. L_4 Strains. J Agroenvironment Sci.

[CR58] Zhang RX, Gong JS, Zhang DD, Su C, Hou YS, Li H, Shi JS, Xu ZH (2016). A metallo-keratinase from a newly isolated *Acinetobacter* sp. R-1 with low collagenase activity and its biotechnological application potential in leather industry. Bioproc Biosyst Eng.

[CR59] Zokaeifar H, Balcázar JL, Saad CR, Kamarudin MS, Sijam K, Arshad A, Nejat N (2012). Effects of *Bacillus subtilis* on the growth performance, digestive enzymes, immune gene expression and disease resistance of white shrimp,Litopenaeus vannamei. Fish Shellfish Immun.

