# Supplementary figures and images for: Recombinant expression and biochemical characterization of a novel keratinase BsKER71 from feather degrading bacterium Bacillus subtilis S1-4
Source: AMB Express. 2020 Jan 15;10:9. doi: 10.1186/s13568-019-0939-6 (PMC6962420; doi:10.1186/s13568-019-0939-6)

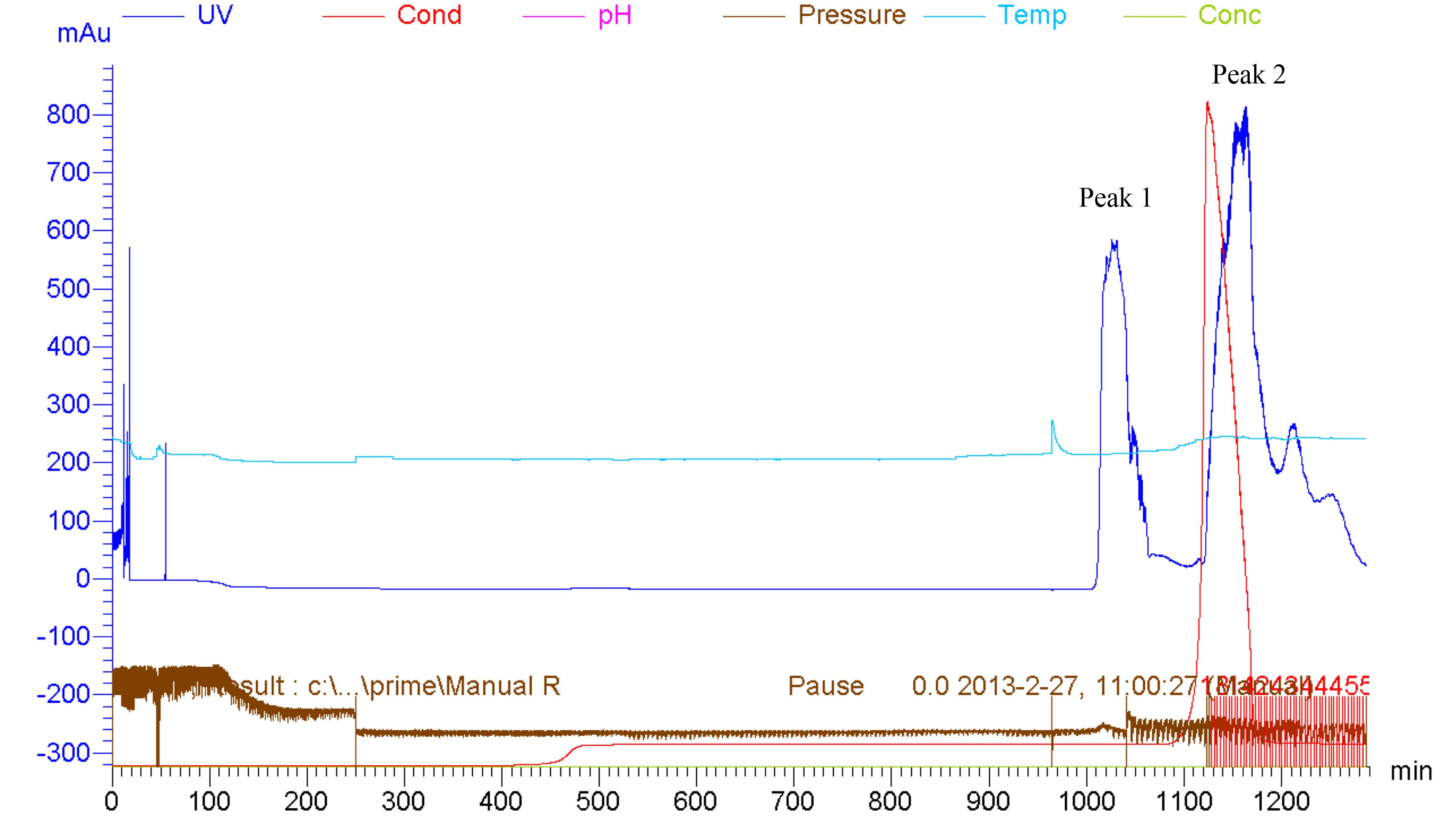

Supplement: Supplementary file 1 — Additional file 1: Figure S1. A typical elution profile of the recombinant keratinase by gel filtration chromatography column. Peak 1: unknown protein; Peak 2: BsKER71 protein; Red peak: ammonium sulfate. [file 13568_2019_939_MOESM1_ESM.tiff]

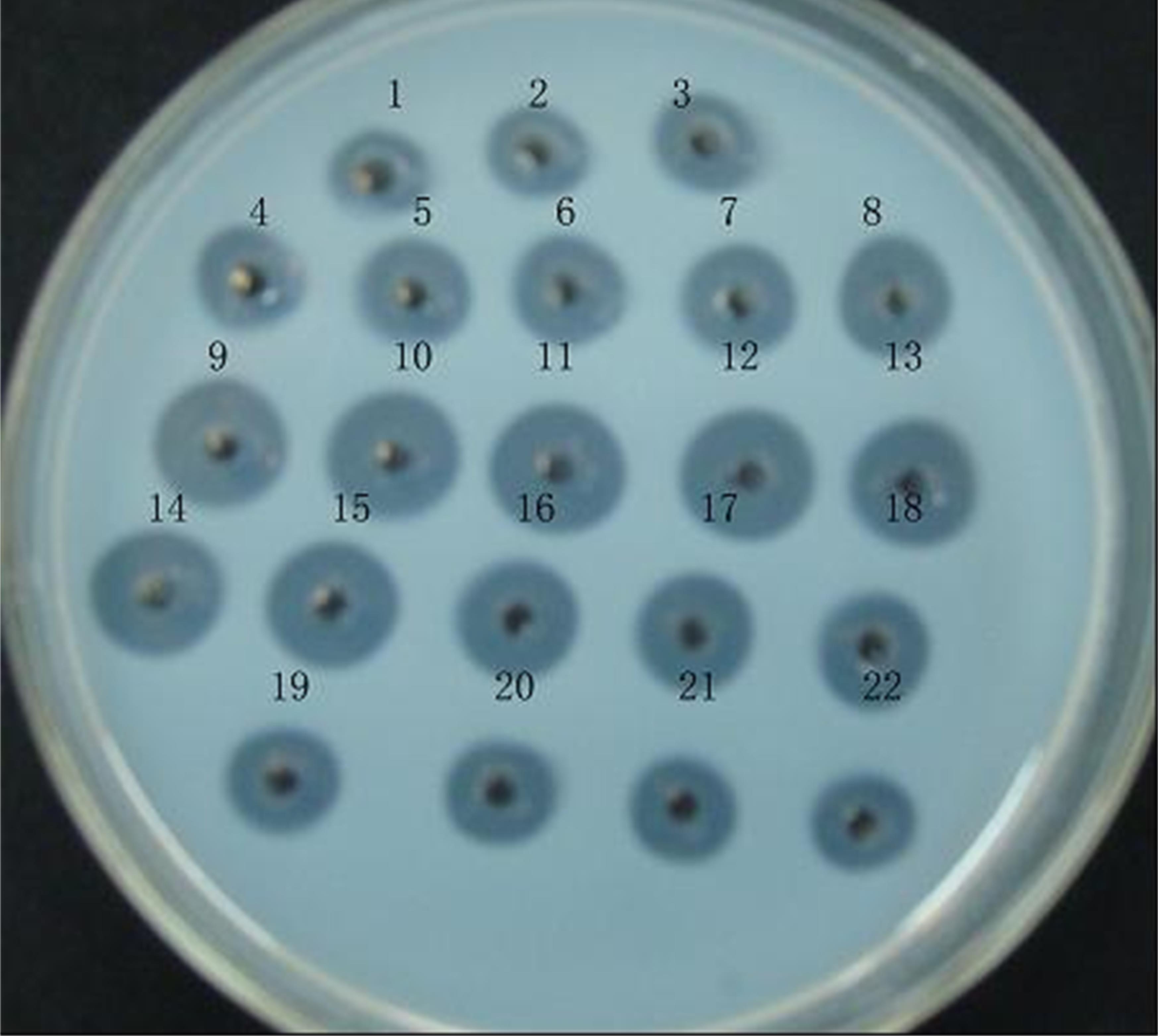

Supplement: Supplementary file 2 — Additional file 2: Figure S2. The protease activity test of eluting samples from peak 2 on the milk containing agarose plate. [file 13568_2019_939_MOESM2_ESM.tiff]

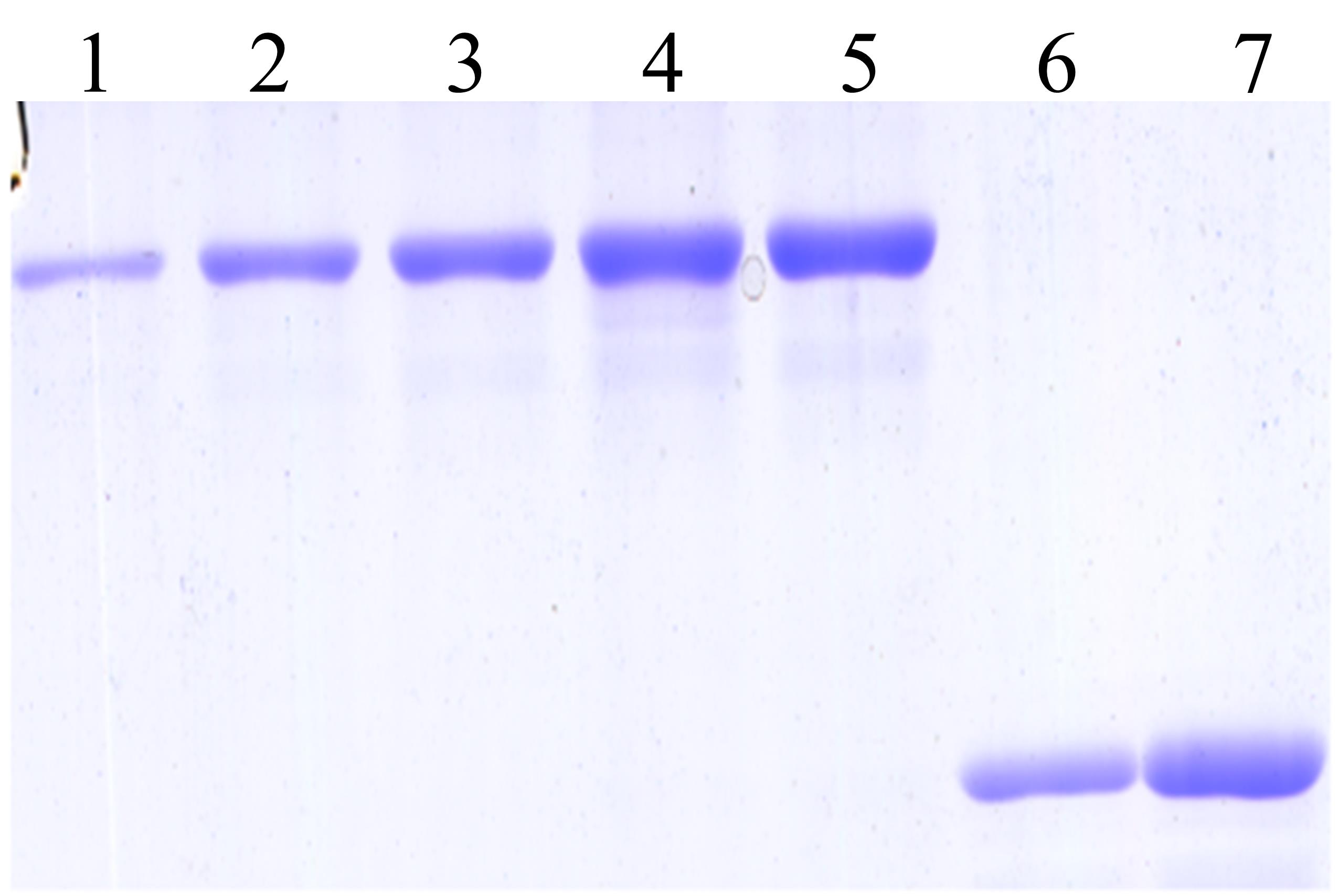

Supplement: Supplementary file 3 — Additional file 3: Figure S3. Quantitative determination of BsKER71 protein on SDS-PAGE. Lane 1, 2, 3, 4, 5 correspond to 0.5, 1.0, 2.0, 3.0, and 4.0 μg BSA, respectively; lane 6 and 7 were loaded with 2.5 μL and 5.0 μL purified BsKER71 protein. [file 13568_2019_939_MOESM3_ESM.tiff]
